# Advancing curation of viral life cycles, host interactions, and therapeutics in Reactome

**DOI:** 10.1128/jvi.02024-24

**Published:** 2025-04-23

**Authors:** Lisa Matthews, Justin Cook, Ralf Stephan, Marija Milacic, Karen Rothfels, Veronica Shamovsky, Bijay Jassal, Robin Haw, Cristoffer Sevilla, Chuqiao Gong, Eliot Ragueneau, Bruce May, Adam Wright, Joel Weiser, Deidre Beavers, Krishna Tiwari, Andrea Senff-Ribeiro, Thawfeek Varusai, Henning Hermjakob, Peter D'Eustachio, Guanming Wu, Lincoln Stein, Marc E. Gillespie

**Affiliations:** 1 Department of Biochemistry and Molecular Pharmacology, NYU Langone Health Center 12297, New York, New York, USA; 2 Department of Molecular Biology and Biochemistry, Simon Fraser University 175075, Burnaby, British Columbia, Canada; 3 Ontario Institute for Cancer Research 90775, Toronto, Ontario, Canada; 4 Institute for Globally Distributed Open Research and Education (IGDORE) 584986, Gothenburg, Sweden; 5 European Molecular Biology Laboratory, European Bioinformatics Institute (EMBL-EBI), Wellcome Genome Campus 497772 , Hinxton, Cambridgeshire, United Kingdom; 6 Medical Informatics and Clinical Epidemiology, School of Medicine, Oregon Health and Science University 6684 , Portland, Oregon, USA; 7 Department of Cell Biology, Universidade Federal do Paraná 28122, Curitiba, Brazil; 8 Department of Molecular Genetics, University of Toronto 204248, Toronto, Ontario, Canada; 9 Department of Pharmaceutical Sciences, College of Pharmacy and Health Sciences, St. John’s University, Queens, New York, USA; Indiana University Bloomington, Bloomington, Indiana, USA

**Keywords:** Reactome, pathway, viral lifecycle, SARS-CoV-2, orthoinference, COVID-19, therapeutic compounds, molecular interactions, community collaboration

## Abstract

Reactome (reactome.org) is a manually curated, peer-reviewed, open-source, open-access pathway knowledgebase of essential human cellular functions. Reactome includes viral life cycles that capture a broad range of virus-induced human pathology. Here, we describe a workflow using collaborative curation strategies, orthoinference procedures, and literature triage to rapidly create reliable molecular models of emergent viruses. The resulting pathway data set rigorously details viral infection pathways, interactions with normal human biological processes, and potential therapeutic compounds.

## INTRODUCTION

Reactome (reactome.org) is a free, open-source, open-access, manually curated, and peer-reviewed pathway knowledgebase that provides a molecular-level blueprint of human cellular physiology (1). This blueprint provides a detailed representation of biomolecular pathways, documenting normal cellular processes and their alterations in disease states. Reactome represents the molecular details of these processes as the properties of the nodes and edges of a graph database. The database provides a flexible and efficient representation of complex biological interactions, enabling researchers to explore the intricate networks of molecular reactions that underpin cellular functions. Reactome’s annotations are meticulously curated by PhD-level scientists and peer-reviewed by field experts, ensuring high-quality data to support translational research and aid in the discovery of clinically actionable targets in various diseases. With its extensive resources, Reactome is a vital tool for researchers, bioinformaticians, scientists, educators, and the general public, facilitating insights into the relationships between genes and the biological processes they drive.

**Fig 1:**
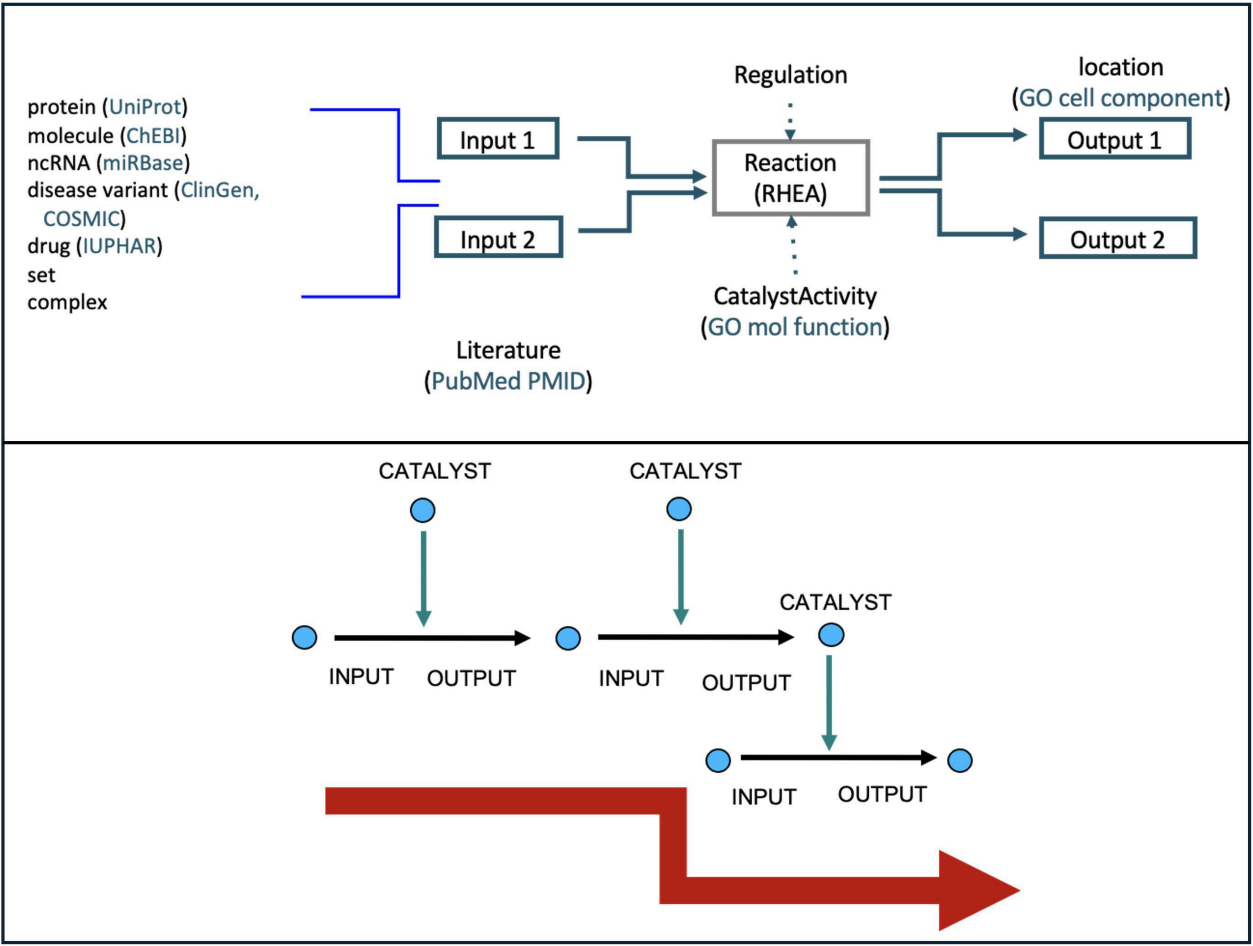
Schematic of Reactome reactions and pathways. (Top) A typical Reactome reaction, including inputs, catalyst activity, and outputs. (Bottom) The stepwise fashion by which larger biological processes can be represented through ordered Reactome reactions.

The core unit of the Reactome data model is the single-step reaction (Fig. 1, top) in which input biochemical (physical) entities are transformed into output ones. Transformations include classic metabolic reactions (e.g., phosphorylation of a substrate by a kinase), transport of a physical entity between subcellular compartments (e.g., the uptake of an extracellular molecule into the cytosol mediated by a transporter), association of two or more physical entities to form a complex, and dissociation of a complex. The catalytic and regulatory roles of gene products in reactions are captured. Physical entities include nucleic acids, proteins, and a simple entity class of smaller biologically relevant molecules, including lipids and sugars, as well as complexes formed of these entities. Causal connections between reactions allow them to be organized into pathways (Fig. 1, bottom). These connections can be direct, e.g., the output of one reaction is the input, catalyst, or regulator of another, or indirect, e.g., genetic evidence shows that disruption of one reaction causes another to fail.

Drug effects that alter protein function are represented in two steps: first, the drug binds the target protein, and then the drug/protein complex regulates the reaction typically catalyzed by the target protein, thereby perturbing the pathway network (2).

Evidence to support all assertions comes from published literature. Reactome biologist curators collaborate with external domain experts to define a consensus view of a process and to identify the key publications that provide evidence for the molecular details of each annotated reaction. This process yields high-quality annotations but scales poorly.

The use of community reference resources supports interoperability. Reactome pathways, reactions, and molecules are cross-referenced to over 100 online bioinformatics resources, including NCBI Gene, Ensembl, UniProt, PubMed, the UCSC Genome Browser, the ChEBI small molecule database, PharmGKB, and IUPHAR/BPS Guide to Pharmacology (GtoPdb) for drugs (2).

Manual inferences from sequence similarity allow the annotation of human proteins that have not been experimentally studied based on the properties of homologous human or model organism proteins (3). Applying the same logic, the orthoinference process has been automated to infer pathway structures for 14 model organisms (4). All non-disease human reactions in Reactome that involve protein participants are eligible for orthoinference, with two exceptions: reactions that were manually inferred from nonhuman data and reactions involving entities from nonhuman species. For each inferred reaction, an equivalent reaction is created for the model organism by replacing each human protein with its model organism homologous protein. This is extended to pathways by inferring a pathway in the model organism for any human pathway for which at least one model organism reaction can be inferred.

Over two decades, Reactome biocuration has assigned, as of edition V91 in September 2024, molecular functions to 11,506 human proteins in 15,492 reactions, assembled into 2,742 pathways. These proteins constitute 58.0% of the 19,846 protein-coding genes predicted in the current (GRCh38.p14) human genome assembly (5). A recent survey suggests that experimental evidence is available for ∼68% of predicted gene products, or 13,500, leaving ∼2,000 annotatable human proteins not yet in Reactome (6). Based on UniProt release 2024_05 (1 October 2024), the viral proteome of human viruses encompasses 6,322 unique, reviewed UniProt entries, of which 2,425 entries are unique viral species-specific proteins, when ignoring viral strains and UniProt entries for which species was not specified. As of the V91 release, Reactome has coverage for 230 unique human viral UniProt entries, in the form of 693 peptides with unique combinations of localization and posttranslational modifications.

This data structure, centered on molecular functions of gene products that enable transformations of physical entities, is well-suited to annotating genetic and infectious diseases. In a genetic disease, a somatic or germline mutant allele encodes an altered gene product that is inactive and fails to mediate its normal reaction or has acquired a novel function and mediates a novel one (7). In an infectious disease, the focus of this paper, an agent such as a virus introduces novel gene products into the human system that enable new reactions to be added to the normal pathway network (e.g., synthesis and assembly of viral components) or modulate normal human ones (e.g., inhibition of human translation factors by viral gene products). This representation reflects the evolution of viruses to co-opt the molecular processes of their hosts to enable their replication. This interface or transition from normal function into the viral disease state is the focus of much clinical research on infectious disease. Reactome captures disease as a comparative set of reactions and pathways that exist in the disease state. This disease state pathway can be compared to the “normal” pathways that exist without the introduction of a disease agent. The difference in the disease state as compared to the normal process details the activation of the immune system and the viral infection strategies that have evolved to avoid these responses.

## VIRAL LIFE CYCLE CURATION

We developed our strategy to annotate viral life cycles over a decade, working on Influenza (2006), HIV (2006), and Cytomegalovirus (2019) (Table 1). We revised, extended, and streamlined this strategy to participate in the community annotation of coronavirus life cycles in response to the 2020 COVID pandemic and are now implementing it to build a crisis-ready set of template viral life cycles. Normally, curators work with a broad literature base, detailing hundreds of experiments that support more than a hundred molecular viral infection reactions. This literature base is accessed with the help of experts in the field who have worked on these infectious agents for decades. These experts guide curators as they choose well-annotated entries in numerous protein, drug, and ontology data sets. In the case of the 2020 COVID pandemic, none of these resources were available to the Reactome Team. Annotation of the SARS-CoV-1 life cycle using pre-pandemic curation methods required 96 days. In comparison, curation of the SARS-CoV-2 life cycle, utilizing the revised approach incorporating orthoinference and automated literature grouping, was completed in 56 days, reducing the total curation time by approximately 42%. The SARS-CoV-2 curation took advantage of a revised streamlined orthoprojection and community annotation strategy that still maintained the rigor and review of the previous curation strategy. Given the dearth of supporting literature, experts, or even known protein participants at the start of curation, this new approach represents a significant success.

**TABLE 1:** Virus life cycles represented in Reactome

Genetic system	Family	Virus - human disease	Reactome
−ssRNA	Paramyxoviridae	Respiratory syncytial virus (RSV) - bronchiolitis	Released doi:10.3180/R-HSA-9820952.1
	Orthomyxoviridae	Influenza A virus - influenza	Released doi:10.3180/REACT_6145.3
+ssRNA	Coronaviridae	SARS-CoV-1 - severe acute respiratory syndrome (SARS)	Released doi:10.3180/R-HSA-9678108.1
	Coronaviridae	SARS-CoV-2 - COVID-19	Released doi:10.3180/R-HSA-9694516.1
	Flaviviridae	Dengue virus (DENV) - dengue fever	Planned
ssRNA-RT	Retroviridae	Human immunodeficiency virus 1 (HIV1) - Lentivirus - AIDS	Released doi:10.3180/REACT_6256.3
dsRNA	Reoviridae	Rotavirus A - diarrheal disease among infants and young children	Ongoing
dsDNA	Herpesviridae	Human cytomegalovirus (HCMV) - subfamily Betaherpesvirinae - mononucleosis	Released doi:10.3180/R-HSA-9609646.1
ssDNA	Parvoviridae	Parvovirus B19 - Fifth disease	Planned

### Viral curation strategy and accelerated curation: the response to COVID

The 2020 COVID-19 pandemic brought together a large, diverse group of bioinformatics researchers who recognized the need for expedited creation of accurate, detailed viral pathway maps to support basic research, variant typing, and drug/therapy optimization (8, 9). The resulting community effort relied on team curation, rapid literature triage, and viral orthoinference projection to extend our viral curation efforts and enhance our ability to efficiently deliver high-quality viral pathway curation to the scientific community.

**Fig 2:**
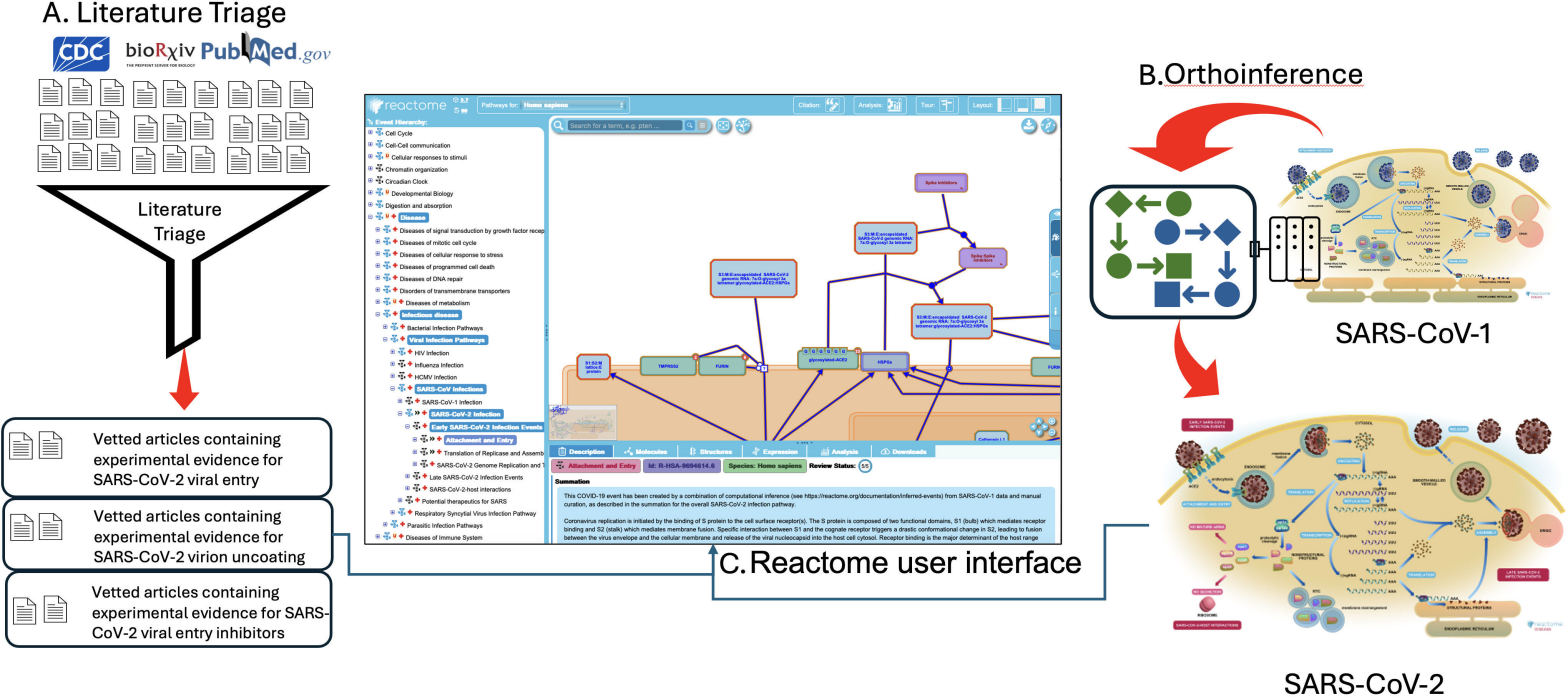
Process by which the literature triage and orthoinference processes were brought together by curators to accelerate manual curation. (**A**) Literature triage and keyword tagging rapidly identified and organized supporting experimental evidence for the events of the SARS-CoV-2 life cycle. (**B**) At the same time, the orthoinference process was used to compute an experimental SARS-CoV-2 life cycle from the complete team-annotated SARS-CoV-1 life cycle. (**C**) The Reactome user interface is composed of three functionally linked panes, a navigable hierarchy (on the left), a primary window containing diagrammatic and entity level views of the pathways (upper right), and an informational pane (lower right). Here, it shows the entity level view of the SARS-CoV-2 virion interacting with TMPRSS2 or Furin upon cellular entry.

In 2020, as expedited curation of SARS-CoV-2 life cycle was needed, the Reactome curatorial approach was augmented with three new approaches (Fig. 2). We used our expert knowledge of other viral life cycles to partition the SARS-CoV-1 life cycle into subpathways that were annotated in parallel and efficiently joined upon completion. The orthoinference process was used to compute an experimental SARS-CoV-2 life cycle from the complete team-annotated SARS-CoV-1 life cycle. Lastly, we used literature triage and keyword tagging to rapidly identify and organize supporting experimental evidence for the events of the SARS-CoV-2 life cycle.

The Reactome effort was further refined by working in parallel with the COVID-19 Disease Mapping project community and the Disease Maps group (10, 11). This broad pathway community annotation approach facilitated access to relevant papers as well as expert reviewers and advisers as SARS-CoV-2-specific published experimental evidence became available.

#### Dividing and conquering the SARS-CoV-1 life cycle

Viral strains and variants must be carefully vetted so that the genomically encoded entities (proteins and nucleic acids) identified in the literature are properly taxonomically attributed in viral life cycle annotations and associated with the appropriate entries from external reference sequence databases. This ensures that virus-specific physical entities (proteins, nucleic acids, and, if applicable, other compounds), as well as the events they participate in, are tagged with the correct viral species and strains. This tagging supports quality-control checks to ensure that species identifiers of the physical entities, events (reactions and pathways) that they participate in, and literature references cited for support are all consistent. We first identified a representative SARS-CoV-1 sequence to be used for annotation (SARS coronavirus Tor2, complete genome NCBI Reference Sequence: NC_004718.3 [https://www.ncbi.nlm.nih.gov/nuccore/30271926]). This strain is well annotated at the genomic and functional level, has the most nearly complete nucleotide sequence, and is commonly used in research labs throughout the world, and substantial peer-reviewed information describing the functions of its proteins was available to support biocuration efforts.

We surmised that we could initially approach the SARS-CoV-1 life cycle, like previously annotated viral life cycles, as five distinct modules—viral attachment and entry into the human cell, translation of viral replicase and assembly of the replication transcription complex, viral genome replication and transcription, translation of viral structural proteins, and virion assembly and release. Two additional modules focused on host-viral interactions and the effects of drugs on the viral life cycle. Curators were assigned to annotate individual modules and to review the curation of adjoining ones, to ensure that possible annotation discrepancies were identified and fixed early in the assembly of the complete life cycle.

#### Orthoinference

Manual annotation of the SARS-CoV-1 pathways was completed in June 2020, in Reactome version 73, at a point when published experimental evidence for the molecular details of the SARS-CoV-2 life cycle was sparse. The published pathway consisted of over 150 reactions describing interactions between the viral proteome and ~140 human proteins and included more than 70 drugs. To create a framework for the evidence-based SARS-CoV-2 annotations, we used a modification of the orthoinference process described above to infer SARS-CoV-2 pathways based on the extensive homology between SARS-CoV-1 and SARS-CoV-2 protein sequences. We selected the SARS-CoV-2 isolate Wuhan-Hu-1 (https://www.ncbi.nlm.nih.gov/nuccore/MN908947) for annotation because it was the first complete genome sequence available. To allow the projection of disease reactions involving host and pathogen entities, both disease and non-disease reactions related to SARS-CoV-1 were considered for orthoinference, as were reactions involving entities from more than one species. In addition, the constraint of having at least one protein participant in a candidate reaction was removed to account for non-protein-containing SARS-CoV-1 reactions such as hydroxychloroquine uptake into cytosolic vesicles. Finally, both protein and RNA entities were inferred from the SARS-CoV-1 infection pathway.

The resulting ortho-predicted SARS-CoV-2 infection pathway events and entities were stored in the curation database and explicitly flagged as computational inferences. As the literature triage process described in the next section yielded data directly describing the functions of SARS-CoV-2 proteins, computationally inferred reactions involving these proteins were checked for accuracy and corrected as necessary. The overall five-module pathway topology, as well as each predicted subpathway and reaction, was reviewed. Revisions included the addition or removal of specific reaction participants and/or events. As literature references describing experimental evidence were added to SARS-CoV-2 reactions, “inferred” flags were removed.

Once complete, the individual sections of the SARS-CoV-2 infection pathway were again internally reviewed by curators. These complete manually revised pathway annotations were then peer-reviewed by external experts, and the reviewers’ feedback was integrated into the final version. The first SARS-CoV-2 infection pathways were first released in September 2020.

#### Literature triage

The challenge in reference selection for the SARS-CoV-2 annotation project in 2020 was identifying, from among the thousands of SARS-CoV-2 papers published mostly as preprints each month, high-quality peer-reviewed references relevant to life-cycle pathway annotation. To address this problem, a “snowballing” computational triaging strategy was developed to review and identify publications appropriate for manual curation.

Several collections of SARS-CoV-2 literature were manually screened with a focus on molecular interactions. These were a Zotero Library built and updated by members of the COVID-19 Disease Mapping project community, CORD-19, LitCOVID, Johns Hopkins literature summary, Cell Press Coronavirus Resource Hub, Nature Coronavirus and COVID-19 updates, and Science’s Latest Coronavirus research (10, 12–17). In addition, two primary literature reference databases, the CDC COVID-19 database and bioRxiv database, were downloaded and automatically text-mined (18, 19).

About 95% of the articles were rejected because they did not contain molecular data relevant to the construction of Reactome’s SARS-CoV-2 life cycle pathway annotations (20). Rejected papers could lack specific enough information, experimental evidence, physical entities, or species information. Those that did advance to the literature selection step were tagged according to the following:

1. Type of publication (e.g., article, review, pre-print, comment)
2. Virus and host species (e.g., SARS-CoV-2, SARS-CoV-1; MERS, ACE2)
3. Entity (specific molecules studied)
4. Methods (e.g., Cryo-EM, ELISA, IC50)
5. Cell line and/or tissue (e.g., vero-E6, lung tissue)
6. Subcellular localization (e.g., plasma membrane, ER)
7. Molecular event (e.g., virus cycle step, pathway, host response)
8. Phenotype (e.g., immune, coagulation)

Reactome curators were assigned sections of the viral life cycle, host-pathogen interaction, or potential therapeutics pathways. Each curator was assisted by an editorial manager and supported by a separate group of triage curators who sorted the rapidly expanding SARS literature.

### SARS-CoV-2 pathway

The combination of multiple curators working in parallel, orthoinference projection, and literature triage accelerated curation while preserving high-quality annotation standards. The initial projection of SARS-CoV-1 to SARS-CoV-2 resulted in 105 CoV-2 reactions from 106 CoV-1 reactions. The first draft of the SARS-CoV-2 pathway was released in September 2020 (Reactome v74). It consisted of 111 reactions involving 489 molecular entities (279 proteins, 12 RNAs, and 198 other entities), supported by citations to 227 publications. Through periodical updates, the pathway was expanded and, as of September 2024, consists of nearly 250 reactions and includes over 300 viral and human proteins and close to 70 drugs, citing over 600 publications.

The SARS-CoV-2 life cycle is represented by five modules, grouped into two pathways, “Early SARS-CoV-2 Infection Events” and “Late SARS-CoV-2 Infection Events.” Interactions of viral and host processes form a separate pathway, “SARS-CoV-2-Host Interactions,” and drug effects are annotated in a pathway, “Potential Therapeutics for SARS” (21–23).

## DISCUSSION: BUILDING A COLLECTION OF REPRESENTATIVE VIRAL LIFE CYCLES

The molecular details of viral life cycles closely parallel normal human processes, facilitating their annotation within the Reactome data model. Specifically, many if not all viral life cycles can be divided into distinct pathway modules, each of which co-opts a distinct normal human cellular process: viral attachment and entry into the human cell (endocytosis), translation of viral replicase and assembly of the replication transcription complex (RNA synthesis and processing), viral genome replication (DNA replication for DNA viruses; templated RNA synthesis for RNA viruses), transcription and translation of viral structural proteins (protein synthesis and processing), and virion assembly and release (exocytosis). Viral processes are thereby integrated into the rich set of basic human biological pathways, including autophagy, the cell cycle, developmental biology, DNA replication, gene expression, innate and adaptive immune systems, metabolism, reproduction, and signal transduction, already annotated in Reactome. The viral infectious disease pathways connect the viral molecular machinery to these human pathways that are activated, altered, or disrupted upon viral infection.

To better support community efforts to deal quickly and effectively with emerging human viral diseases, we aim to build a collection of representative viral life cycles, to be used as templates to annotate and analyze new viral disease processes exactly as we used SARS-CoV-1 to create a projected SARS-CoV-2 pathway. The choice of key representative viruses for curation (Table 1) is driven by the global impact of known viral pathogens, organized to provide reliable virus family coverage within the Baltimore system, (24) and refined using consideration of expert collaborator availability and high-quality viral genomic data (25).

A key requirement of this strategy is the ability to reliably infer a plausible life cycle for an emerging virus from the known life cycle of a well-studied relative. Here, we have described the steps required to do this for coronavirus, working from a SARS-CoV-1 template to SARS-CoV-2. We have demonstrated the utility of an automated literature mining and tagging system to efficiently validate a computationally inferred viral life cycle as experimental evidence emerges, highlighting poorly understood aspects of the life cycle for experimental analysis on the one hand and identifying potential drug targets within well-studied areas on the other. Common features of viral life cycles allow the annotation process to be modularized and completed in parallel by multiple curators.

Together, all these pieces support the assembly of a collection of viral life cycles modeled as Reactome pathways that capture a broad range of virus-induced human pathology and that serve as templates for the rapid creation of reliable models of the behavior of emergent variant strains of these viruses.

Reactome provides curated pathway data and computational tools for analyzing virus-host interactions, immune responses, and disease mechanisms. Reactome pathways have been used to study how SARS-CoV-2, Influenza, HIV, and other viruses manipulate host cellular processes. Pathway Analysis Tools analyze transcriptomics, proteomics, and metabolomics data. Researchers regularly upload gene and protein data sets to identify host pathways that change or emerge during viral infection. The Reactome curation strategy is driven by comparative analysis across viral species infection and replication strategies. This approach ensures that Reactome has assembled representative viral life cycles that cover the breadth of viral genome type (dsDNA, ssDNA, dsRNA, and ssRNA), genome organization, replication strategy, virion structure, and assembly mechanism. Reactome enables the study of common and unique host responses across different virus life cycles, identifying conserved viral strategies and potential broad-spectrum antiviral targets.

By mapping virus-host interactions onto human biological pathways, researchers identify potential drug targets or existing drugs that can be repurposed. Reactome integrates data from the IUPHAR/BPS Guide to Pharmacology (25), identifying existing drugs that may modulate key pathways affected by viral infection. The drug activity or potential activity is directly linked to the molecular process and viral infection step (26). Groups have used the Reactome data set to identify lead compounds that target the SARS-CoV-2 replication machinery and explore combinatorial therapies and new target identification (9, 27).

Reactome’s extensive immunological pathway data for humans is projected into 14 evolutionarily divergent eukaryotic species. The human immune system data is connected to the annotated viral life cycles. This data allows for extensive investigation of the molecular events that occur in the host immune response, including insights into cytokine signaling, T-cell and B-cell activation, and interferon responses (28, 29).

These studies take advantage of network-based analysis and system biology approaches integrating Reactome network biology tools with viral and human pathway data to study virus-induced molecular networks. These approaches provide researchers with a map of the systemic effects of viral infection (30–32).

Reactome pathways include therapeutic compounds and detail the molecular entities that they interact with and functionally alter. A data set that rigorously details the integration of basic human biological processes, therapeutic compounds, and viral infectious molecular pathways is a powerful tool driving research. The ongoing expansion of viral pathway content and community collaboration is driving the construction of a viral infection knowledge base that serves as a powerful tool for rapidly predicting clinical behavior and drug sensitivities of emerging pathogenic viruses.
